# An Adaptive 3D Neighbor Discovery and Tracking Algorithm in Battlefield Flying Ad Hoc Networks with Directional Antennas

**DOI:** 10.3390/s24175655

**Published:** 2024-08-30

**Authors:** Yunjie Yuan, Gongye Ren, Xingyu Cai, Xuguang Li

**Affiliations:** 1 Xi’an Electronic Engineering Research Institute of China, Xi’an 710100, China; rengongye@126.com (G.R.);; 2 Institute of Computer Application Technology, Norinco Group, Beijing 100089, China

**Keywords:** adaptive 3D neighbor discovery, beam tracking, directional antennas, 3D scanning, flying ad hoc networks, battlefield

## Abstract

Neighbor discovery and tracking with directional antennas in flying ad hoc networks (FANETs) is a challenging issue because of dispersed node distribution and irregular maneuvers in three-dimensional (3D) space. In this paper, we propose an adaptive 3D neighbor discovery and tracking algorithm in battlefield FANETs with directional antennas. With time synchronization, a flying node transmits/receives the neighbor discovery packets sequentially in each beam around it to execute a two-way handshake for neighbor discovery. The transmitting or receiving status of each discovery slot depends on the binary code corresponding to the identification of the node. Discovered neighbor nodes exchange their 3D positions in tracking slots periodically for node tracking, and the maximum tracking period is determined by node velocity, beamwidth, and the minimum distance between nodes. By configuring the relevant parameters, the proposed algorithm can also apply to two-dimensional planar ad hoc networks. The simulation results suggest that the proposed algorithm can achieve shorter neighbor discovery time and longer link survival time in comparison with the random scanning algorithm in scenarios with narrow beamwidth and wide moving area. When the frame length increases, the protocol overhead decreases but the average neighbor discovery time increases. The suitable frame length should be determined based on the network range, node count, beamwidth, and node mobility characteristics.

## 1. Introduction

In current battlefield scenarios, omnidirectional antennas can provide signal reachability within a single hop range for communication equipment with unknown and time-varying relative position relationships based on the omnidirectional coverage characteristics of transmitted and received signals to neighboring nodes. Omnidirectional antennas are widely used in distributed networking communication equipment, such as ultra-short wave radio stations, microwave broadband ad hoc networks, etc. [1]. However, with the continuous improvement in communication requirements in the aspects of covertness, anti-interference, communication distance, data rate, networking scale, and network throughput in the new generation of three-dimensional battlefield environments on land, the communication functions and communication performance based on omnidirectional antennas present the following shortcomings [2]:

- Poor concealment and weak anti-interference ability: the coverage of the signal is much larger than the area of the receivers, which is susceptible to reconnaissance and interference conducted by the enemy. Omnidirectional antennas have no resistance to directional interference.
- Short communication distance and low speed: the gain in omnidirectional antennas is low, and blindly increasing transmit power is unfavorable in terms of power consumption, heat dissipation, cost factors, etc., resulting in limited communication distance and communication rate. In addition, signal quality is easily affected by terrain and electromagnetic environments and is consequently unstable.
- Low network capacity and high collision rate: the omnidirectional coverage of the signal inhibits the possibility of space division multiplexing between adjacent nodes, and the network throughput cannot be improved. The probability of inter-node collision increases with the increase in channel access density when the network is overloaded.

In contrast to omnidirectional antennas, the phased array antenna, as an important type of directional antenna, has a flexible beamforming ability, and its transmit power, antenna gain, beam direction, beamwidth, and beam shape can be configured flexibly on demand, which brings a significant improvement in the performance of communication distance, communication rate, anti-reconnaissance, anti-interference, and network capacity [3]. The phased array antenna constitutes a promising technical trend for the new generation of battlefield intelligent broadband communication.

Compared with communication using omnidirectional antennas, which does not need to know the position relationship between the transmitter and the receiver, narrow-beam communication with directional antennas needs to know the relative position relationship between the transmitter and the receiver in advance. The beam control parameters can be calculated based on relative positions, and the beams of the transmitter and the receiver are aligned during the communication period to make full use of high antenna gains brought by phased array antennas.

In tactical mobile ad hoc networks with directional antennas, because of the absence of central node coordination and the unknown and time-varying position relationship between nodes, it is necessary to design a neighbor discovery and tracking strategy to obtain the relative position relationship between neighbors in real time and create the conditions for beam alignment. This strategy is the prerequisite for the operation of multiple access, routing, and data transmission in narrow-beam distributed ad hoc networks, and it is among the essential technologies for communications based on phased array antennas.

In recent years, following the advancement in manufacturing technologies for unmanned aerial vehicles (UAVs) and their reduced cost, UAVs have been widely used in both civil applications and military applications [4,5]. In military application scenarios, various electronic devices have been mounted on UAVs to implement remote surveillance, reconnaissance, and armed attack, which has been demonstrated as an important measure in modern warfare. In addition, UAVs can form distributed backbone networks, known as flying ad hoc networks (FANETs), to provide communication services for ground users, from individual soldiers to panzers. In this paradigm, phased array antennas can be equipped to realize the communication distance over hundreds of kilometers with the data rate over hundreds of megabits per second [6,7]. Furthermore, the employment of directional antennas on UAVs can also improve the ability to resist eavesdropping and jamming, which is crucial in the battlefield. Currently, the communications between UAVs generally rely on ground control stations or satellite relays, and the scalability of UAV networks is constrained. In the FANETs, however, UAVs can directly communicate with their neighbors, or transmit data packets to remote UAVs depending on relay UAVs. Because of their bright prospects, FANETs with directional antennas have gained the interest of industrial and academic communities. Because of the agile mobility and extensive mobility range in three-dimensional (3D) space, the neighbor discovery and tracking strategy in FANETs with directional antennas is much more complicated than that in planar ad hoc networks.

There are a lot of studies on neighbor discovery and node tracking in two-dimensional (2D) ad hoc networks with directional antennas. The authors of [8] propose two novel decentralized and low-complexity reinforcement learning-based directional neighbor discovery schemes. Neighbor discovery is mapped to a stochastic multi-player game, where each node independently adjusts its policy via the Q-learning-based scheme to minimize the discovery latency. Hong et al. [9] propose oblivious multi-antenna neighbor discovery algorithms in airborne networks based on the Chinese Remainder Theorem. The proposed algorithms do not require any central control to achieve beam rendezvous under oblivious circumstances. The authors of [10] propose a neighbor discovery protocol with directional antennas when time is not synchronized. When neighboring nodes construct their working state sequence according to their identifications (IDs) and scan based on intended angular speed, neighbor discovery can be completed within a certain period of time. In [11], a Q-learning-based directional neighbor discovery algorithm is designed to improve the traditional blind algorithm. The Q-learning-based neighbor discovery consists of the following stages: the initial stage for acquiring initial location information, the reinforcement learning stage for determining the working mode and antenna direction, and the completion stage for implementing mutual discovery. Sui et al. [12] apply four reinforcement learning algorithms to neighbor discovery in ad hoc networks with directional antennas. The simulation results demonstrate that reinforcement learning-based neighbor discovery algorithms can improve the efficiency of neighbor discovery, especially in the case of a one-way handshake. The authors of [13] propose a cognitive framework to adjust the reception probability to minimize the neighbor discovery time in internet of thing networks with directional antennas. The dynamic programming method is utilized to calculate optimal reception probabilities. Zhang et al. [14] propose a Multi-token Sector Antenna Neighbor Discovery (M-SAND) protocol to enhance the efficiency of neighbor discovery in asynchronous directional ad hoc networks. The master token holders and multiple slave token holders can simultaneously discover their neighbors without mutual interference. The authors of [15] introduce successive interference cancellation technology into directional neighbor discovery algorithms to unpack multiple collision packets by distinguishing multiple packets in the power domain. With successive interference cancellation technology, the collided neighbor discovery packets can be demodulated correctly, and the neighbor discovery time is reduced. In [16], directional neighbor discovery is formulated as a Markov multi-armed bandit (MAB) problem. A three-way handshake algorithm with collision feedback is proposed in order to better perceive collisions in the network. The authors of [17] establish a theoretical framework for the asynchronous neighbor discovery problem. They propose a beam scan sequence that guarantees two nodes in the neighborhood can discover each other even if they are equipped with heterogeneous directional antennas. The aforementioned references are aimed at neighbor discovery problems in 2D ad hoc networks with directional antennas. Regarding the application of FANETs, plenty of studies focus on this topic in 3D ad hoc networks with directional antennas. The authors of [18] study the neighbor discovery problem between high-speed aircraft and low-speed ships and propose a Limited Random Algorithm that makes use of approximate location information. Khan et al. [19] propose a 3D scanning method for establishing line-of-sight links between UAVs. Two types of antennas are equipped on the UAV. A directional antenna, which is mounted on a mechanically steerable spherical structure/head, is used for data transmission, and an omnidirectional antenna is used for time synchronization. In [20], a novel two-way neighbor discovery algorithm is proposed for FANETs, and the height of UAVs is considered to follow a Gaussian distribution. The 3D spherical space is divided into the following subspaces: upper spherical crown, middle spherical band, and lower spherical crown, and the transmission directions of hello packets are chosen probabilistically based on the subspaces. In [21], the drones randomly choose a circular scanning path and angular speed and perform a three-way handshake to discover each other. The drones change the circular scanning path and angular speed if the discovery is not successful within an optimal time interval. Song et al. [22] propose a 3D directional multi-channel neighbor discovery algorithm in mmWave FANETs through cross-layer optimization of both the physical layer and medium access control (MAC) layer. With a hybrid beamforming architecture, reply packets are transmitted on multiple channels simultaneously, which reduces the neighbor discovery time. The authors of [23] consider the impact of earth curvature on beam steering in large-scale FANETs and propose a partitioned beam-scanning scheme based on the altitude of scanning nodes. Chen et al. [24] formulate the 3D directional neighbor discovery problem and derive the worst-case discovery delay bound. Guided by the theoretical results, the authors design a distributed neighbor discovery algorithm, achieving the minimal worst-case discovery delay based on the Chinese Remainder Theorem.

All the mentioned 3D directional neighbor discovery strategies apply to specific scenarios or UAVs with specific hardware configurations, and they are not adaptive to application scenarios or hardware configurations. For example, the algorithm proposed in [18] is suitable for mutual discovery between high-speed aircraft and low-speed ships, but it cannot be used for neighbor discovery between UAVs. Another example [22] considers hybrid beamforming architecture for multi-channel reception on UAVs, which is not practical for most types of UAVs. In addition, neighbor discovery is a basic function of multi-access protocols, and it is necessary to integrate neighbor discovery into the design of multi-access protocols. Based on the facts above, in this paper, we propose an adaptive 3D neighbor discovery and tracking algorithm in FANETs with directional antennas. With time being synchronized via an on-board global navigation satellite system (GNSS) receiver, it provides an efficient and continuous way to obtain the neighbor node information in the range of one hop for communication equipment with a multi-planar or circular phased array or other types of directional antennas. On this basis, a time division multiple access (TDMA) multi-access protocol is proposed, which provides the whole working principle from neighbor discovery and node tracking to data transmission reservation based on tracking. Then, aiming to build a phased array antenna-based ad hoc communication system, based on the relevant baseband signal processing, RF transceiver channel, phased array antenna, and other physical layer parameters, the relevant parameters of the multi-access protocol are designed. Finally, the network access delay, tracking success rate, protocol cost, and other metrics of the multi-access protocol under different network sizes, different node distributions and motion properties, and different beamwidth are simulated, and the results of functional verification and performance analysis are presented. The protocol design framework and the adaptation of relevant parameters are provided for subsequent engineering prototype development. The main features that distinguish our proposal from the existing works are summarized as follows:

- Our algorithm can be adapted to various scenarios; it is not restricted to 3D space: Though our algorithm is designed for 3D neighbor discovery and tracking, it can be used in 2D space with minor revisions. In addition, our algorithm can be adapted to a wide range of node mobility, from static networks to fast speeds up to 800 km/h.
- Our algorithm can be configured for given purposes: The frame length, the number of discovery time slots and tracking time slots in a frame, the length of binary sequences of node IDs, etc., can be modified to achieve different design purposes, such as short neighbor discovery delay, high tracking success rate, and low protocol overhead.
- Our algorithm integrates neighbor discovery into the multi-access protocol: To evaluate the impact of neighbor discovery on multi-access protocols, we design an integral multi-access protocol that includes three basic functions: neighbor discovery, node tracking, and data transmission. The interaction among these functions is analyzed via simulation.
- Our algorithm imposes a few restrictions on antenna types: The phased array antennas are considered in this paper as an instance of directional antennas, but this is not the only case. Horn antennas, reflector antennas, and lens antennas can all be equipped on UAVs to realize direct transmission and reception with proper arrangement and mechanically steerable structures.

The main contributions of this paper are as follows:

- A method is proposed to determine the sending and receiving state of each node in neighbor node discovery. Based on this method, the average time of neighbor node discovery increases slowly with the number of nodes in the one-hop range, which has the advantages of low overhead and exemption from central node coordination.
- A method to determine the tracking period based on node mobility and minimum distance between nodes is proposed, and a dynamic reservation method for data transmission time slots based on periodic tracking is designed, which can improve the utilization rate of channel resources and decrease the data transmission delay.
- System-level simulation is carried out to evaluate the performance of the proposed algorithm. Based on relevant physical layer parameters and system workflow, the detailed design of the multi-access protocol proposed in this paper is carried out based on node distribution characteristics, motion characteristics, and service transmission requirements of the equipment in multiple typical battlefield scenarios. Through system-level simulation, the practical value of the application of this protocol in vehicle-mounted and airborne platforms is verified.

The rest of this paper is organized as follows. Section 2 introduces the design guideline of the neighbor node information acquisition strategy applied to phased array antenna-based FANETs and describes and analyzes related parameters. Section 3 presents the neighbor node discovery and tracking strategy for obtaining neighbor node information in 3D FANETs. Section 4 presents the TDMA multi-access protocol based on the strategy described in Section 3 and the method for setting related parameters. Section 5 verifies the performance of the proposed neighbor discovery and tracking algorithm in multiple typical scenarios and analyzes the results. Finally, we conclude this paper in Section 6. Table 1 provides a list of acronyms used in this paper, and Table 2 provides a list of symbols used in this paper.

## 2. Design Guideline

The information acquisition of a neighbor in phased array antenna-based ad hoc networks is defined as searching and discovering the neighboring nodes in the one-hop communication range, obtaining the real-time relative position relationship for them, and using this information for the beam alignment required for data transmission.

In order to achieve the above goals, it is necessary to design a neighboring node information acquisition strategy and then determine the relevant parameters based on the actual scenarios. The strategy mainly includes a scanning strategy and a tracking strategy, and related parameters include beamwidth and various period lengths.

### 2.1. Scanning Strategy

Transmitting and receiving beam alignment means that the two nodes in the transmitting and receiving states point the beam at each other in the same time period. Therefore, the core of neighbor discovery in FANETs is to direct the two neighboring nodes distributed in the 3D space to work in the transmitting and receiving states during the same period of time and to align the beam directions with each other under the condition that there is no central node coordination.

The occurrence of the above events requires the node to periodically traverse the one-hop communication range in 3D space through a certain scanning mode. The scanning mode controls the beam scanning sequence and the sending or receiving status in each beam during neighbor discovery. Its design goal is to update the neighbor relationship in the one-hop communication range as quickly as possible. The opportune update of the neighbor relationship gives rise to timely recognition of topology changes, and the neighbor nodes can be tracked constantly.

Different distributions of neighboring nodes determine different one-hop communication ranges. Taking the general FANETs in 3D space as an example, the one-hop communication range of a node is the whole sphere with itself as the center and the one-hop communication distance as the radius. In mobile ad hoc networks (MANETs) of ground vehicle-mounted platforms, the one-hop communication range of a node is a ring-shaped space with a certain thickness with itself as the center of the circle and the one-hop communication distance as the radius. In the ground-to-air access networking scenario, the one-hop communication range of the aerial node is a cone with itself as the vertex, the one-hop communication distance as the slant height, and the one-hop communication range of the ground node is an inverted cone with itself as the vertex and the one-hop communication distance is the slant height. Different beam scanning ranges bring about different protocol overheads.

### 2.2. Tracking Strategy

No matter what scanning policy is adopted, the one-hop communication range must be traversed in the neighbor discovery, and the nodes both in the sending or receiving state cannot discover each other. Therefore, the average delay of neighbor discovery based on a narrow beam is much higher than that based on omnidirectional antennas. If each data transmission depends on the neighbor discovery, a large amount of data to be sent may be accumulated, resulting in serious delay and packet loss.

After the neighbor discovery period, once a neighbor node is found, the coverage of the beam of this node and the neighbor node will overlap for a period of time, during which the immediate relative position relationship can be used for data transmission. If the update of the relative position relationship can be completed within this period and the update actions are implemented periodically, the one-hop accessibility with this neighbor node can be maintained without waiting for the next neighbor discovery period.

Based on the above requirements, by periodically obtaining the latest relative position relationship within the effective coverage period of each beam, the data transmission requirements between neighboring nodes can be continuously satisfied. Although relative displacement may occur between nodes during the cycle, they are still within the spatial coverage range of the original beams.

### 2.3. Beamwidth

The narrower the scanning beamwidth, the smaller the coverage area, resulting in the need for more beams to complete 3D space scanning, and the longer it takes for neighbor discovery. On the contrary, the wider the beamwidth of the scanning beam, the fewer beams are needed to complete 3D space scanning, and the neighbor discovery is faster, but the probability of multiple neighboring nodes covered by a single scanning beam increases, which results in conflicts and brings low neighbor discovery efficiency.

For tracking, factors such as a too-narrow tracking beamwidth, too close of a distance between neighboring nodes, and high relative mobility will lead to tracking failure because neighboring nodes can easily run out of their current beam coverage. This problem can be avoided by increasing the tracking frequency, but a higher tracking frequency will bring higher protocol overhead. There should be a compromise between these factors.

In addition, the beamwidth determines the transmit–receive gain, which in turn affects the communication distance. The wider the beamwidth, the smaller the single-hop communication distance, and the number of hops required for end-to-end communication may increase, resulting in increased transmission delay. In addition, the communication distance of neighbor discovery and neighbor tracking should be as consistent as possible and less than or equal to the communication distance of data transmission. Data transmission is usually adaptive to multiple modulation modes to obtain the highest communication rate at the current channel quality. At the same transmit power, a higher communication rate usually corresponds to a smaller communication distance.

### 2.4. Cyclical Settings

#### 2.4.1. Neighbor Discovery Cycle

The design of the neighbor discovery cycle involves the following three factors. Firstly, it is necessary to complete mutual discovery with all neighboring nodes within a hop range in as short a time as possible. Secondly, an ad hoc network supports entry and exit for nodes at any time, which means the discovery of neighboring nodes should not only exist at the beginning of network construction but also run through the whole process of network operation. Thirdly, if the neighbor discovery process is too centralized, there may be no opportunities for the discovered neighboring node tracking, leading to data packet accumulation, which can cause discovery failure and data transmission timeout.

Therefore, the discovery process should be dispersed and interlaced with neighbor tracking and data transmission. The dispersion density should be calculated with parameters such as average and maximum network access delay, data throughput requirement, etc.

#### 2.4.2. Neighbor Tracking Cycle

The neighbor tracking cycle is strongly correlated with the relative mobility, the minimum distance between the transmitter and receiver, and the beamwidth. The higher the relative mobility, the smaller the distance, and the narrower the beamwidth. Thus, the corresponding tracking cycle should be shorter, and vice versa.

Meanwhile, when the quality of the wireless channel is unstable, the tracking frequency should be increased to ensure the tracking success rate. This results in a shorter tracking cycle and increased protocol overhead.

## 3. Neighbor Information Acquisition Strategy

### 3.1. Overview

The process of neighbor information acquisition and data transmission can be described as follows: After the node is turned on, it periodically performs neighbor discovery and neighbor tracking. Data transmission is scheduled with neighbor tracking and is carried out during time periods other than neighbor discovery and neighbor tracking.

The neighbor discovery method is as follows: Each node performs a 3D space scan for multi-round in the transmitting or receiving state. In each scanning round, the nodes in the transmitting and receiving states act as the discovery initiator and discovery responder, respectively. The initiator sends its identity, position, and tracking slot status on each beam. The responder listens on each beam. If the responder receives the information, it reserves and replies the available tracking opportunity to the initiator. Through this interaction, the initiator and the responder can also obtain their relative position relationship. The main results are summarized as follows:

- In each scanning round, the node in the transmitting state will discover all nodes in the receiving state, and the node in the receiving state will be discovered by all nodes in the sending state.
- After a multi-round of scanning, all nodes in the network will experience the mutual exchange of transmitting and receiving states and finally complete mutual discovery with all neighboring nodes within its one-hop range.

The following methods are adopted for neighbor tracking: Both tracking parties act as a tracking initiator and a tracking responder and point their beams at each other according to the state used and the relative position obtained from the last neighboring discovery or the last neighboring tracking. The initiator sends its identity, position, data transmission demand, and data transmission time slot status. The responder listens. If the responder receives the information, it reserves and replies the available data transmission opportunity to the initiator. Through this interaction, the initiator and the responder can also update their relative position relationship. The main results are summarized as follows:

- Once the tracking is successful, the node will continuously update the information of the relative position relationship with the discovered neighbor nodes to ensure that the beam direction information can be used when there is a demand for data transmission.
- If the tracking fails, the node can only re-establish the neighbor relationship based on the next neighbor discovery, which usually takes much longer time than the next neighbor tracking. Data transmission is not available until that time comes.

There are two ways to obtain the relative position relationship. Method 1: The initiator and the responder calculate the relative position relationship by exchanging their position information, which can be acquired from the platform. Method 2: The initiator and responder use their own phased array antenna to calculate the angle of the coming wave direction. The second method does not need position information from the platform and has additional requirements for RF channel and physical layer frame format design, while the first method is the opposite. Both methods can be used depending on actual engineering needs, which is beyond the scope of this paper.

### 3.2. Scanning Mode for Each Round in Neighbor Discovery

**Figure 1:**
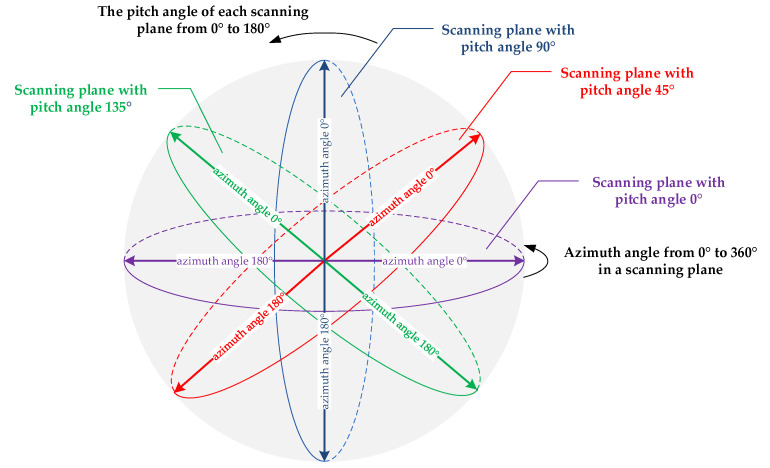
Beam scanning principle of the adjacent node discovery period. In the ENU coordinate system with the origin at the node, the scanning plane with a pitch angle of 0° is the x-y plane. If the node is in the transmitting state, it transmits probe packets from the east and scans in each beam position in this plane clockwise. If the node is in the receiving state, it attempts to receive probe packets from the west and scans in each beam position in this plane clockwise. After all the beams in this plane are scanned, the node scans in another plane with a pitch angle of, say, φ (φ is the pitch beamwidth). That is, the scanning plane rotates along the y-axis with φ, from x positive direction to z positive direction. Multiple scanning planes rotating along the y-axis can cover the 3D spherical space.

For each round of sending or receiving scanning, the node completes the scanning within the whole sphere with itself as the center and the one-hop communication distance as the radius. The scanning range in the azimuth and pitch are [0°, 360°] and [0°, 180°], respectively. For each scanning plane, the node completes its scanning in azimuth [0°, 360°), and the one-hop scanning is composed of multi-plane traversing in pitch direction [0°, 180°), as shown in Figure 1.

We define the following parameters:

- θ is the azimuth beamwidth;
- φ is the pitch beamwidth.

As shown in Figure 1, it takes 360/θ beams to traverse the [0°, 360°) range and takes 180/φ beams to traverse the [0°, 180°) range. In each scanning plane, all nodes located in the scanning plane experience at least one beam alignment after 360/θ beam scanning. After going through 180/φ scanning planes, all nodes located in the one-hop coverage experience at least one beam alignment.

In each scanning plane, all nodes scan in a clockwise direction. Nodes in the transmitting state start from a unified direction (such as 0°), and nodes in the receiving state start from a direction 180° more than the first direction (such as 180°). All nodes adopt an anticlockwise direction from the negative direction of y axis in the ENU coordinate system, starting from pitch angle 0° and ending at pitch angle 180°, to traverse all the scanning planes.

If the number of beams required for each scanning round is n2, then n2=360/θ×180/φ.

### 3.3. Determination of Transmitting and Receiving States in Different Scanning Rounds in Neighbor Discovery

In each scanning round, the state of transmitting or receiving remains unchanged. Each node determines its state for each scanning round according to its ID.

The ID is a natural number in the range [1, the maximum number of nodes in the network (i.e., network scale)]. By converting the ID of each node into binary code, the transmitting and receiving states of this node in each scanning round can be obtained as follows: ‘1’ and ‘0’ in the binary code represent the transmitting and receiving states of the node, respectively. The binary codes must comply with the principle that the numbers of ‘1’ and ‘0’ values should be the same, while the distribution of ‘1’ and ‘0’ values should be distinguishing. The number of bits in the binary code is the number of scanning rounds.

Let the number of scanning rounds be n1. By making the network size ≤${\;C}_{n1}^{floor(n1/2)}$, we can obtain n1 from the minimum value satisfying the formula. For example, when the network size is 40, n1 is 8, and the numbers of ‘1’ and ‘0’ values in each binary code is four. Since $C_{8}^{4}=70$, that is, the network size can be supported up to 70, the IDs of these 40 nodes can be randomly assigned in the range [1, 70].

It can be seen that the larger the size of the network, the more scanning rounds are required for neighbor discovery within one-hop coverage. Table 3, Table 4 and Table 5 show the value of n1 when the maximum network size is 3, 6, and 10, and the transmitting and receiving state for each ID in each scanning round can also be found in these tables.

Table 6 lists the n1 required for different network scales. When the actual number of nodes in the network is smaller than the network scales indicated in Table 6, the required n1 for the network is shown in Table 6. It can be seen that n1 increases much more slowly than the network scale. For example, when the network size increases from 20 to 252, the number of scanning rounds required only doubles.

After n1 rounds of scanning, all nodes in the network have experienced the reciprocity of transmitting and receiving states between pairs.

## 4. Multiple Access Protocol

### 4.1. TDMA Frame Format

All nodes in the network adopt the unified time frame format. Based on time synchronization, each node in the network can obtain the position of current time in the unified time frame, allowing the node to operate this TDMA frame format for multiple access.

**Figure 2:**
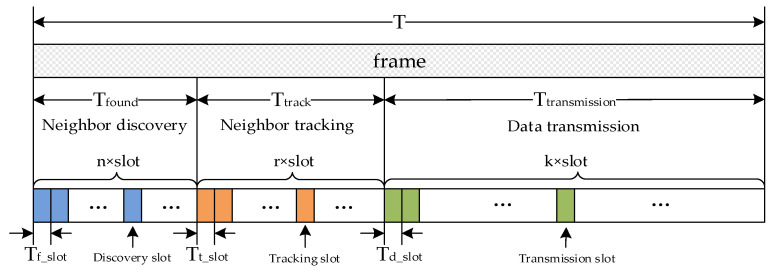
Theoretical TDMA frame format.

The TDMA frame is composed of the following sub-phases: neighbor discovery, neighbor tracking, and data transmission. Each sub-phase is composed of time slots, as shown in Figure 2.

We define the following parameters:

- T is the duration of the frame;
- $T_{found}$ is the duration of the discovery phase in a frame;
- $T_{track}$ is the duration of the tracking phase in a frame;
- $T_{transmission}$ is the duration of the data transmission phase in a frame;
- $T_{f\_slot}$ is the duration of the discovery slot;
- $T_{t\_slot}$ is the duration of the tracking slot;
- $T_{d\_slot}$ is the duration of the data transmission slot;
- n is the number of discovery slots in a frame;
- r is the number of tracking slots in a frame;
- k is the number of data transmission slots in a frame;
- z is the number of subframes in a frame.

The functions of each phase are as follows:

- Neighbor discovery phase: used to obtain the relative position between nodes and tracking slot reservation.
- Neighbor tracking phase: used to update the relative position between nodes and reserve the data transmission slot on the premise of successful discovery.
- Data transmission phase: used for data transmission between nodes on the premise of successful tracking.

**Figure 3:**
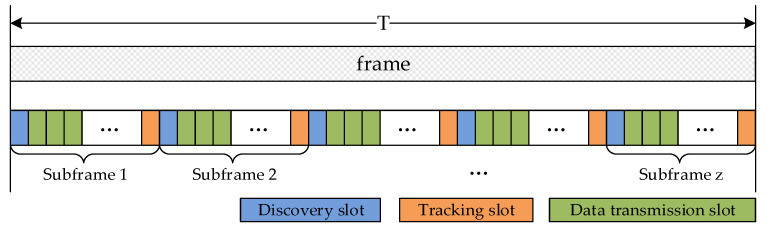
Actual TDMA frame format.

Based on the theoretical format of the frame in Figure 2, as we mentioned before, the discovery slot, tracking slot, and data transmission slot should be dispersed in the entire frame. Because each frame contains $z=min\left\{n,r\right\}$ subframes and the composition of each subframe is the same, each subframe contains n (n≥0) discovery slots, r (r≥0) tracking slots, and k (k≥0) data transmission slots, as shown in Figure 3.

Based on the slot distribution principle shown in Figure 3, tracking slot reservation and data transmission slot reservation follow the following rules:

- Rule 1. If a node successfully discovers a neighboring node in a discovery slot, it reserves the nearest and idle tracking slot for itself and the neighboring node.
- Rule 2. If a node successfully tracks in the tracking slot, it books the same tracking slot in the next frame, and, at the same time, according to the data transmission requirements with the neighboring node, it reserves data transmission slots closest to the current time.
- Rule 3. The reserved data slots should distribute as continuously as possible. Data transmission needs should be given priority to the initiator and then to the responder. The upper limit of the data slot number reserved is used to maintain fairness. The subframe of the last reserved data slot should be no later than the same subframe in the next frame.
- Rule 4. Before data transmission slot reservation, the node calculates the transmission delay, the propagation delay, and the processing delay for aggregated data packets. From the total delay, we can obtain the number of data transmission slots required for reservation.

### 4.2. Parameter Settings

The neighbor nodes that have discovered each other need to be tracked periodically for relative position relationship updating. Once the tracking fails, the neighbor nodes need to be discovered again, which may require a long time. The closer the nodes are, the shorter the time it takes to move out of the coverage at the same speed, which will cause tracking failure. Therefore, the tracking time interval required by the closest distance between two nodes should be taken as the unified tracking cycle for the whole network. In this scheme, two nodes need to complete tracking at least once in each frame, that is, the tracking cycle equals the frame length.

**Figure 4:**
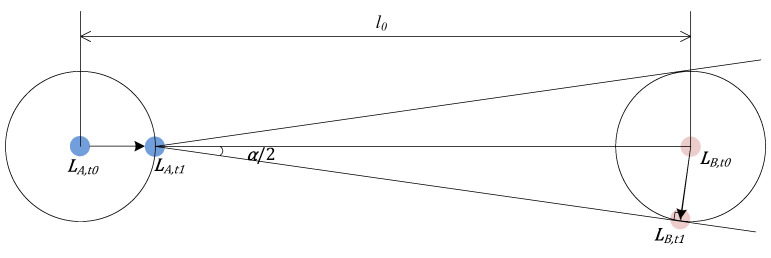
Changes in the relative positions of nodes due to mobility.

Let A and B be two nodes whose tracking cycle is T, as shown in Figure 4. At time t0, A and B send each other their positions, denoted $L_{A.t0}$ and $L_{B.t0}$, respectively. At time t1 (t1−t0=T), A takes B’s position at time t0 as B’s position at time t1, and B takes A’s position at time t0 as A’s position at time t1. Therefore, A points its beam at $L_{B.t0}$ (based on its current position $L_{A.t0}$), and B points its beam at $L_{A.t0}$ (based on its current position $L_{B.t0}$). Let the maximum moving speed of both nodes be v, the distance between A and B at time t0 be $l_{0}$ (this is the minimum distance for node tracking, if the distance between A and B is less than $l_{0}$, the node tracking function will fail), and the beamwidth be α (take the smaller values of θ and φ). The most extreme case is shown in Figure 4, when $L_{A.t1}$ and $L_{B.t1}$ are located on the ring (the radius of the circle is vT), when A moves towards $L_{B.t0}$, the space for B in the α range is minimal. In this case, $\frac{vT}{l_{0}-vT}=\sin\frac{\alpha }{2}$, and then T is obtained, that is, the tracking cycle between A and B cannot be greater than T. When A and B track again, they interact with their respective new positions $L_{A.t1}$ and $L_{B.t1}$, and the process is repeated at the next tracking moment t2.

$T_{f\_slot}$, $T_{t\_slot}$, and $T_{d\_slot}$ are calculated with the actual packet length, physical layer modulation mode, and maximum distance between nodes. To facilitate protocol design and engineering implementation, it is recommended to unify the values of these three parameters.

The tracking sub-phase duration $T_{track}$ is determined by the tracking slot duration $T_{t-slot}$ and the tracking slot number r. r relates to the maximum number of neighboring nodes. Since each node should be allowed a chance to be tracked once by all neighboring nodes and to track all neighboring nodes actively once in a single frame, $r=2\times \left(c_{m}-1\right)$, $T_{track}=T_{t\_slot}\times r$, where $c_{m}$ is the maximum number of neighboring nodes. If the design is carried out for $c_{m}\leq 20$, r≥38, and the actual value can be 40.

When T and $T_{track}$ are constant, the duration of neighbor discovery $T_{found}$ and the duration of data transmission $T_{transmission}$ trade off each other. The longer the $T_{found}$, the higher the number of discovery slots n in each frame, and the faster a node enters the network, but a shorter $T_{transmission}$ results in fewer data slots k in each frame, making the throughput decrease. On the contrary, the shorter the $T_{found}$, the slower a node enters the network, but the throughput is improved, and the network will be more efficient. This paper adopts the strategy of first determining $T_{found}$ based on the requirements of the network access delay and then judging whether $T_{transmission}$ meets this requirement. According to the neighbor information acquisition strategy, the mutual discovery in the one-hop coverage can be completed after n1×n2 time slots. When the maximum number of neighboring nodes is 20, n1=6. Let θ=6°, φ=9°, and consider the total scanning range in the pitch direction as 180°; then, n2=360°/θ×180°/φ=120, and n1×n2=720.

When n=r=40, a maximum of 720/40=18 frames are needed to complete discovery with all neighboring nodes, and the maximum network access delay, in this case, is 18×T. Compare this delay with the network access delay required, if the requirement is met, n can be 40, and each frame contains $z=min\left\{n,r\right\}=40$ subframes.

## 5. Simulation Results

This section aims to verify the performance of the proposed adaptive 3D neighbor discovery and tracking algorithm in FANETs with directional antennas. The network simulation is carried out on various scenarios with different network ranges, node counts, node distributions, and node mobility characteristics, and performance metrics such as neighbor discovery time, link survival time, and accumulated link offline time are obtained. Then, the performance analysis of related protocol parameters in different scenarios are presented.

### 5.1. Simulation Platform and Simulation Model

**Figure 5:**
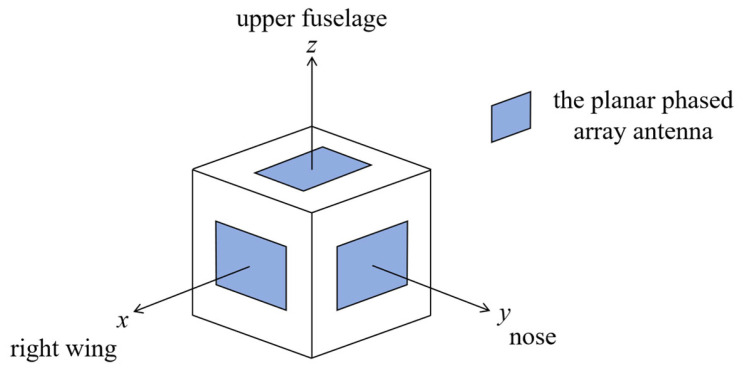
The mounting positions of the planar phased array antennas on the UAV. The other three planar phased array antennas are blocked and not shown.

**Figure 6:**
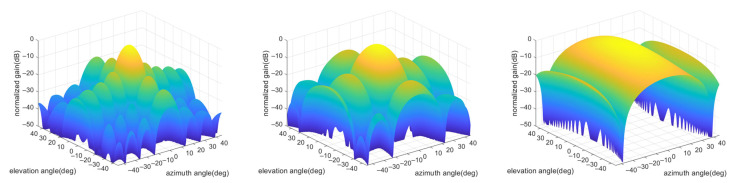
The normalized antenna patterns with beamwidths of 6° × 9° (**left**), 12° × 18° (**middle**), and 24° × 36°(**right**), with the beamwidth in the azimuth direction ahead and the beamwidth in the elevation direction afterward. The array sizes of these antenna patterns are 12 × 8 (**left**), 7 × 5 (**middle**), and 4 × 2 (**right**), with the number of elements in the azimuth direction ahead and the number of elements in the elevation direction afterward. The beam direction in all three figures is the normal direction. When the beam direction deviates from the normal direction, the beamwidth widens, and the antenna gain drops accordingly.

The simulation is conducted using a professional network simulator OPNET. This software is easy to use and is widely employed in network performance evaluation. OPNET contains a suite of networking modeling techniques, from the physical layer characteristics configured in the pipeline stage to the network topology defined in the network model. The simulation model of this work can be partitioned into five parts as follows:

- Network model: The network range, the number of nodes, node distribution, and node trajectory are defined in the network model. These configurations are set according to specific scenarios, as shown in Section 5.2.
- Node model: The node model defines the function of each node in the network. Since we concentrate on neighbor discovery and tracking in this paper, the modules for the network layer and higher layer functions are not included. The designed node model includes a MAC module, a transmitter module, a receiver module, and an antenna module. The MAC module controls the transmitting or receiving state and the beam direction, constructs the transmitted packets, and handles the received packets. The transmitter module and the receiver module set the parameters related to physical layer technologies, such as the radio frequency, bandwidth, data rate, and modulation method.
- Process model: The process model defines the function of each module in the node model. The function of each module is defined via the finite state machine and programs. The construction of the MAC process model is the main effort in this simulation, which directs the nodes to operate according to the proposed algorithm.
- Pipeline stage: The pipeline stage defines the processing flow of packets from transmitters to receivers. The functions of the pipeline stage include calculating transmission delay and propagation delay, determining the validity of the received packets, calculating the received power according to antenna models, etc.
- Antenna model: To evaluate the impact of phased array antennas accurately, we calculate the antenna gain based on the mathematical model instead of the default antenna model in OPNET. In order to transmit or receive signals in any direction in the 3D space, the UAV is modeled as a cube with each face mounted on a planar phased array antenna, as shown in Figure 5. The planar antennas operate in Ku-band and share the same size, with 12 elements in the azimuth direction and 8 elements in the elevation direction. The normalized antenna patterns with different beamwidths are shown in Figure 6. The coverage of each planar antenna is ±45° in the azimuth direction and ±45° in the elevation direction, and any direction can be caught by one of these antennas no matter how the UAV rotates. When the UAV flies along the defined trajectory, its attitude changes continuously and can be obtained from the inertial navigation system. Given its own attitude information, described by yaw, roll, and pitch, together with its own global coordinate and that of the peer node, the UAV can determine which planar antenna is oriented to the peer UAV and corresponding azimuth and elevation angles through coordinate transformation [25]. For ground vehicles, if communicating with UAVs is not relevant, only four planar antennas are mounted at four horizontal orientations to cover the horizontal 360° ring-shaped space.

### 5.2. Simulation Scenario Design and Parameter Settings

In this paper, the following three typical battlefield scenarios are considered:

- Scenario 1: Networking among stationary ground vehicles in air defense scenarios at key locations;
- Scenario 2: Networking among mobile ground vehicles in mobile battlefield scenarios with armored vehicles;
- Scenario 3: Aerial inter-aircraft networking in cooperative battlefield scenarios with unmanned platforms such as fixed-wing UAVs and rotary-wing UAVs.

Each scenario is subdivided into multiple specific scenarios according to node count, node mobility, distribution range, and distribution characteristics. Table 7 elaborates on each scenario. Although our neighbor discovery and tracking algorithm is aimed at 3D FANETs, it can also be applied in 2D MANETs with minor revisions. In 2D MANETs, the spherical scanning space in Figure 1 degenerates into a scanning plane with a pitch angle of0° to lower the overhead of neighbor discovery. In order to demonstrate the versatility of the proposed algorithm, in addition to 3D FANET scenarios, we also conduct the simulation in planar stationary ad hoc networks and MANETs.

**Figure 7:**
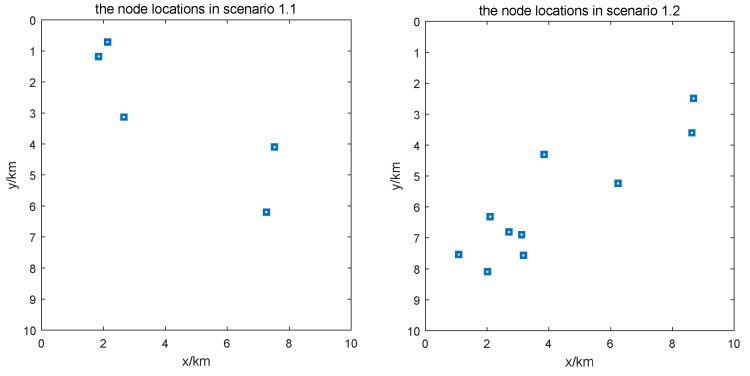
Node distributions for simulation in scenarios 1.1 (**left**) and 1.2 (**right**).

The node distributions for simulation in scenarios 1.1 and 1.2 are shown in Figure 7.

**Figure 8:**
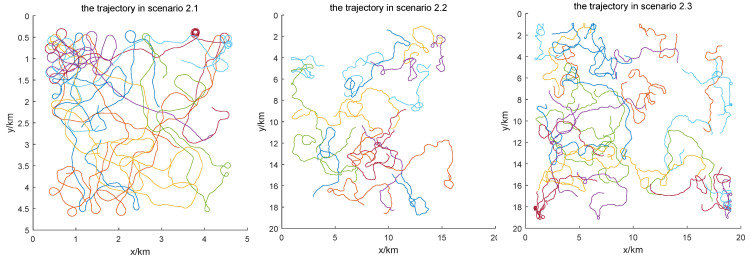
The trajectories of nodes in scenarios 2.1 (**left**), 2.2 (**middle**), and 2.3 (**right**). All the trajectories contain 1000 points, resulting in a duration of 16 min and 40 s. Each colored line represents the trajectory of a node.

**Figure 9:**
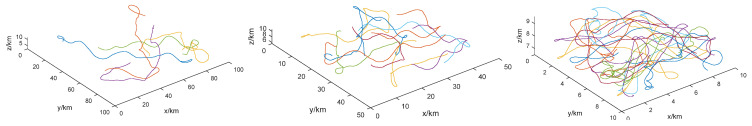
The trajectories of nodes in scenarios 3.1 (**left**), 3.2 (**middle**), and 3.3 (**right**). All the trajectories contain 1000 points, resulting in a duration of 16 min and 40 s. The altitude range in these scenarios is from 6 km to 10 km. Each colored line represents the trajectory of a node.

In order to simulate the real trajectory of mobile nodes, this paper builds a mobility model based on the mobility model proposed in [26]. The mobility model contains the following parameters: moving range, upper and lower limits of the moving speed, steering angle, upper and lower limits of the pitch angle, minimum distance between nodes, etc. The trajectory generated by this model contains a sequence of points, and the node moves along the straight line between successive points. The time interval between successive points is 1 s in this simulation. When the node enters the region near the boundary, the node is forced to turn to make it move towards the center region. In each time interval, the node randomly chooses to turn left or right and move upward or downward, and it continuously adjusts the speed of movement in the defined range. In addition, the yaw angle, roll angle, and pitch angle are calculated according to the moving direction of the node. According to the proposed mobility model, the trajectories of nodes in scenarios 2.1, 2.2, and 2.3 are shown in Figure 8, and the trajectories of nodes in scenarios 3.1, 3.2 and 3.3 are shown in Figure 9.

According to the method presented in Section 4.2, the maximum tracking periods required with different beamwidths in each scenario are calculated, as shown in Table 8. As the beamwidth becomes wider, the maximum tracking period becomes larger, and more time can be left for data transmission in a frame. Since the beamwidth in the normal direction is the minimum beamwidth among all directions, the maximum tracking period calculated based on the normal beamwidth applies to beamwidths in other directions.

According to the node count in each scenario in Table 7, the value of n1 is obtained, as shown in Table 9. To set the value of n2, we need to determine the beamwidth in each scenario. If a narrow beam is employed, the communication distance is large but numerous beams must be used to cover the entire 3D spherical space, which increases the neighbor discovery delay. Otherwise, if a wide beam is employed, the communication distance is short, and a moderate number of beams is enough to cover the entire 3D spherical space. Considering the network range in different scenarios and the communication distance of the three kinds of beamwidths, the appropriate beamwidth is selected for each scenario, and the corresponding value n2 is obtained, as shown in Table 9. The beamwidth used in scenarios 1.1, 1.2, 2.1, and 3.3 is 24° × 36°, and the network ranges of these scenarios are no more than 10 km × 10 km. The beamwidth used in scenarios 2.2, 2.3, and 3.2 is 12° × 18°, and the network ranges of these scenarios are no more than 50 km × 50 km. For scenario 3.1 with the largest network range, the narrowest beamwidth should be used to realize large-distance communication.

The proposed neighbor discovery and tracking procedures contain two-way packet transmission. Thus, a single time slot should accommodate the sum of the transmission delay (for the probe packet and the replied packet), two-way propagation delay (considering the farthest distance between the transmitting and receiving nodes), the modulation and demodulation delay (for the two packets), the processing delay (for the two packets), and so on. The length of the time slot, $T_{slot}$, designed for different scenarios is shown in Table 9.

In this protocol, the frame length equals the tracking period. In order to ensure the tracking success rate under the condition of maximum relative mobility, the frame length should be less than the maximum tracking period corresponding to the selected beamwidth. Under this premise, in order to reduce the tracking cost, the frame length, T, is chosen to be as large a value as possible so that is not larger than the maximum tracking period while facilitating the choice of other related parameters. Based on the above principles, the values of T in different scenarios are obtained, as shown in Table 9. After T and $T_{slot}$ are determined, the values of n, r, and k are determined accordingly, as shown in Table 9. Note that $n+r+k=T/T_{slot}$.

### 5.3. Simulation Results and Analysis

According to Table 9, the settings of T, $T_{slot}$, n, r, k, n1, and n2 are correlated. When $T_{slot}$, n, r, n1, and n2 are constant, k increases with the increase in T, the protocol overhead decreases, and the average neighbor discovery time increases. To simplify the simulation while demonstrating the impact of the principal parameters on the performance, we fix the values of $T_{slot}$, n, r, n1, and n2 in Table 9. Then, we observe the changes in the average neighbor discovery time, the average link survival time, and the accumulated link offline time in each scenario by changing T and verify the rationality of the selected T values shown in Table 9 in each scenario. The link survival time refers to the length of time during which the neighbor relationship is maintained with the neighbor node after a node successfully discovers it. Within the link survival time, data transmission is feasible between the node and the neighbor node. The accumulated link offline time refers to the accumulated time during which the link is not connected between any pair of nodes during the simulation period. When a link is re-established, the current link offline time is added to the accumulated link offline time.

**Figure 10:**
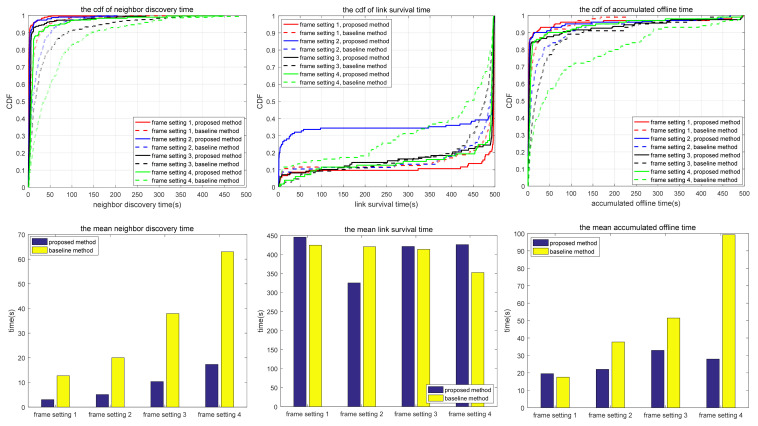
Neighbor discovery time, link survival time, and accumulated link offline time in simulation scenario 1.1.

**Figure 11:**
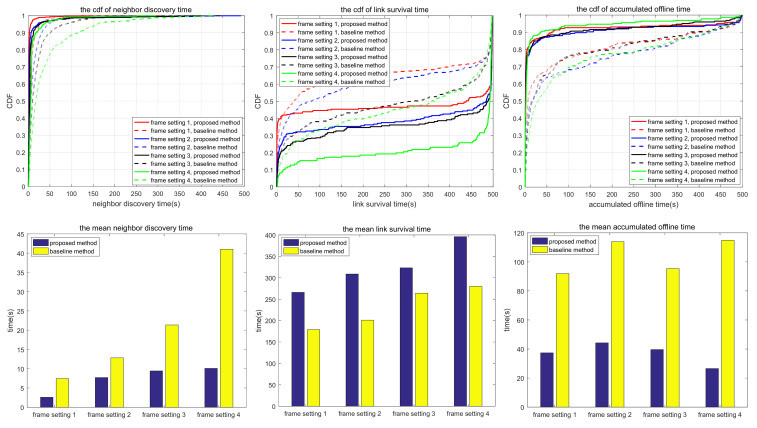
Neighbor discovery time, link survival time, and accumulated link offline time in simulation scenario 1.2.

**Figure 12:**
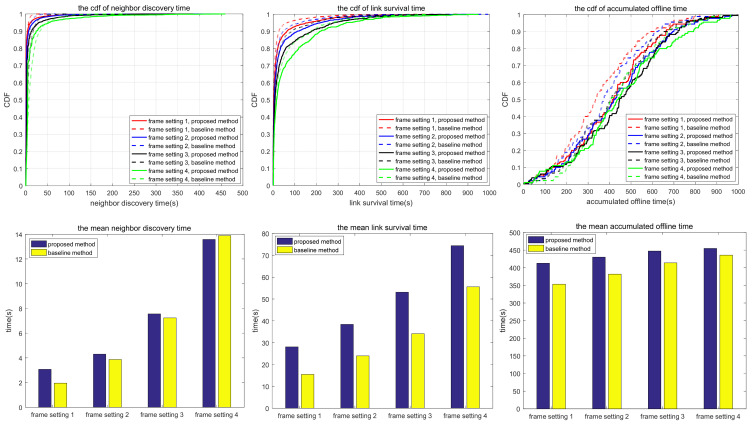
Neighbor discovery time, link survival time, and accumulated link offline time in simulation scenario 2.1.

**Figure 13:**
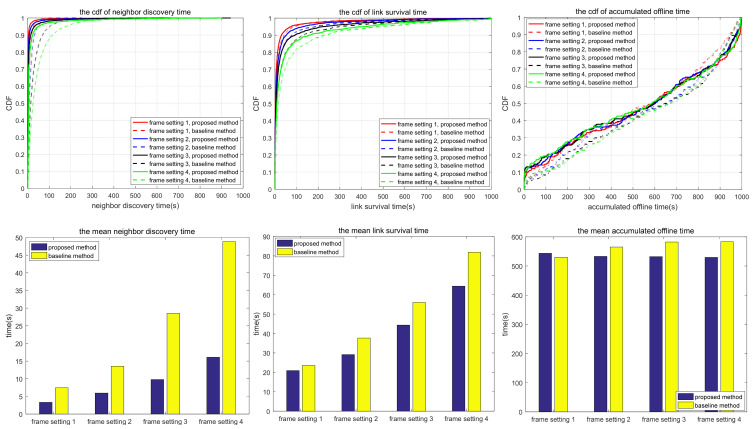
Neighbor discovery time, link survival time, and accumulated link offline time in simulation scenario 2.2.

**Figure 14:**
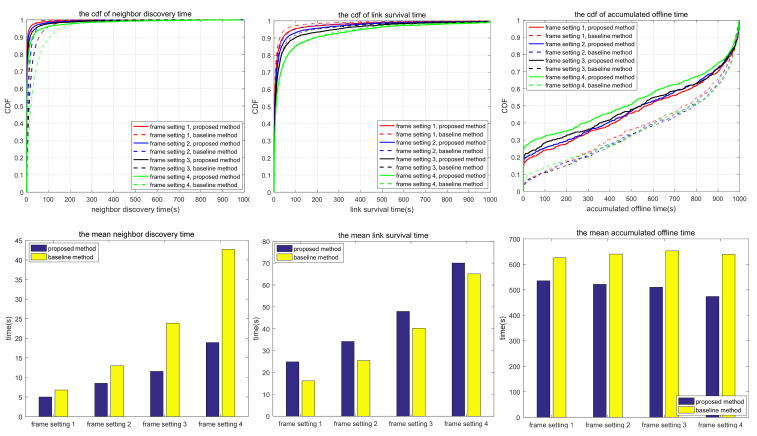
Neighbor discovery time, link survival time, and accumulated link offline time in simulation scenario 2.3.

**Figure 15:**
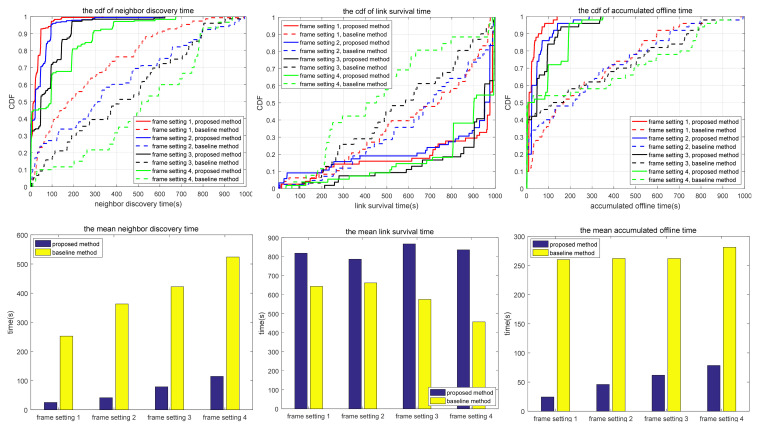
Neighbor discovery time, link survival time, and accumulated link offline time in simulation scenario 3.1.

**Figure 16:**
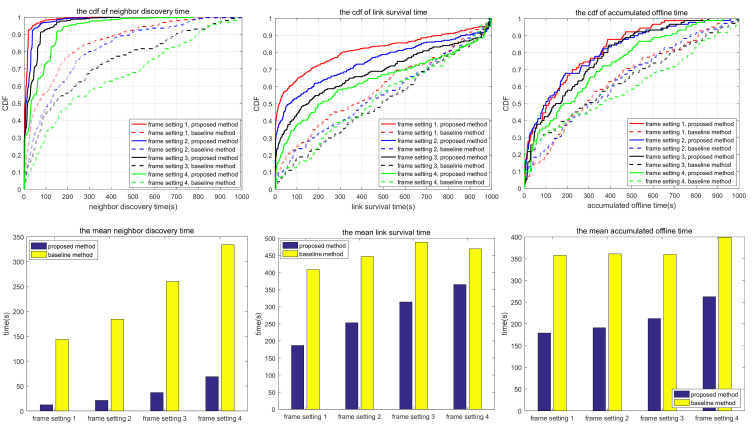
Neighbor discovery time, link survival time, and accumulated link offline time in simulation scenario 3.2.

**Figure 17:**
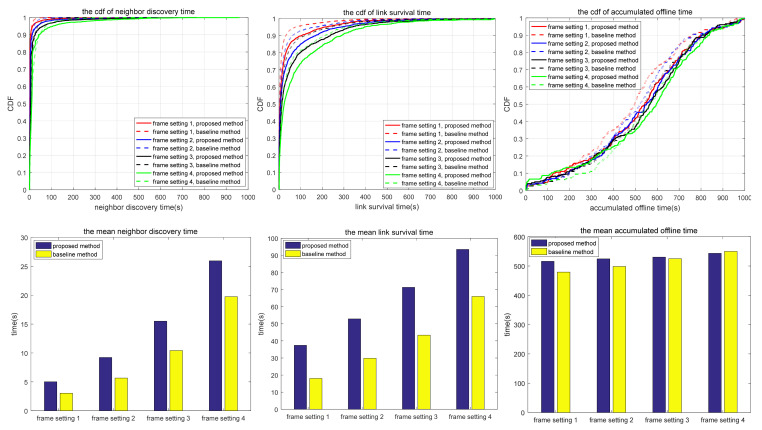
Neighbor discovery time, link survival time, and accumulated link offline time in simulation scenario 3.3.

The simulation also compares the random scanning mode with the neighbor node information acquisition scanning proposed in this paper, which is used to analyze the adaptability of different scanning modes to different scenarios. In the random scanning mode, each node randomly selects the sending and receiving states and beam direction in the discovery slot. In scenarios 1.1 to 1.2 and 2.1 to 2.3, the scanning is implemented in 2D space, while in scenarios 3.1 to 3.3, the scanning is implemented in 3D space. T and protocol overhead in each scenario (including neighbor node discovery overhead and neighbor node tracking overhead, which are the total overhead of the multi-access protocol proposed in this paper) are shown in Table 10, and the simulation results are shown in Figure 10, Figure 11, Figure 12, Figure 13, Figure 14, Figure 15, Figure 16 and Figure 17. In Table 10, frame setting 2 corresponds to the T value set in Table 9, frame setting 1 is halved based on frame setting 2, and frame setting 3 and frame setting 4 are doubled successively. The simulation duration of scenario 1.1 and scenario 1.2 is 500 s, and the simulation duration of the other scenarios is 1000 s.

As shown in Figure 10, in terms of neighbor discovery time, the neighbor discovery time of the proposed method is smaller than that of the comparison method under the four frame structures, and it increases with the increase in frame length. However, for some special node distributions (such as multiple nodes lying along a line), in the proposed method, multiple nodes send discovery request packets at the same time, resulting in a demodulation failure at the receiving node, so packet interaction in the discovery phase cannot be completed. As a result of this phenomenon, some nodes never discover specific neighbor nodes, and the average number of undiscovered nodes is shown in Table 11. While in the comparison method, the probability of this occurrence is greatly reduced. In terms of link survival time, there is no difference between the proposed method and the comparison method. In the static scenario, the link survival time is mainly affected by interference and random attenuation. In this scenario, a beam with a beamwidth of 24° × 36° is used to send and receive signals, and the node distribution is relatively close. It is usual that a node is interfered with by other sending and receiving nodes in the tracking process. In terms of the accumulated link offline time, the proposed method is smaller than the comparison method under the four frame structures. This is because the proposed method can rediscover neighboring nodes more quickly after the link is broken. In summary, in a shorter frame structure, the frequency of neighbor discovery and node tracking is higher, resulting in a shorter neighbor discovery time and accumulated link offline time, but the time overhead for network management is correspondingly increased.

Compared with scenario 1.1, scenario 1.2 has more nodes, but the other configurations remain unchanged. As shown in Figure 11, similar to scenario 1.1, compared with the comparison method, the proposed method has a shorter neighbor discovery time, longer link survival time, and shorter accumulated link offline time. With the increase in T, both the neighbor discovery time and link survival time of the proposed method increase, but the accumulated link offline time has no obvious change rule. Compared with scenario 1.1, each frame in scenario 1.2 contains 20 tracking time slots. The probability that neighboring links choose the same tracking time slot is lower, and the probability of mutual interference is lower, resulting in a lower probability of link disconnection. As shown in Table 11, the average number of undiscovered nodes in the proposed method increases sharply compared with scenario 1. This is because more nodes lead to specific interference scenarios under fixed node topology, and the use of a beam with a beamwidth of 24° × 36° makes the interference more serious. The proposed method may not be able to find specific neighbors in static topology, which can be solved by frequency hopping, narrow-beam communications, or upper-layer routing protocols.

As can be seen in Figure 12, in terms of neighbor discovery time, there is little difference between the proposed method and the comparison method in scenario 2.1. This is because the nodes continue to move in a small range and the signals are received and sent by a wide beam, in which case the sequential neighbor discovery scanning mode has little advantage. In terms of link survival time, the proposed method is superior to the comparison method because the comparison method has a shorter neighbor discovery time under most frame settings, so it is easy to establish bidirectional links. In the same scenario, if tracking loss occurs in the comparison method, the bidirectional link will be broken. In the proposed method, after a unidirectional link is broken, there is a reverse link to maintain the link between nodes, thus increasing the link survival time. In terms of accumulated link offline time, there is little difference between the proposed method and the comparison method because the movement of nodes reduces the possibility of interference in the case of sequential scanning, so the average number of undiscovered neighbors in the proposed method is greatly reduced compared with the static scenario, as shown in Table 11.

Scenario 2.2 has a larger network scope and more nodes than scenario 2.1. As shown in Figure 13, the neighbor discovery time of the proposed method is shorter than that of the comparison method, but there is little difference in link survival time and accumulated link offline time. Under special moving trajectories, two nodes may not find each other when scanning according to the proposed method, but random scanning can avoid this problem, as shown in Table 11.

Compared with scenario 2.2, scenario 2.3 has more nodes, but the other configurations remain unchanged. As shown in Figure 14, the neighbor discovery time, link survival time, and accumulated link offline time of the proposed method are superior to those of the comparison method. Similar to scenario 2.2, the proposed method may fail to discover some neighbors. The proposed method has an average neighbor discovery time of 8.49 s in frame setting 2 (protocol overhead 33.33%) in scenario 2.3, and 3.31 s in frame setting 1 (protocol overhead 40%) in scenario 2.2. The average neighbor discovery time in frame setting 2 (protocol overhead 20%) in scenario 2.2 is 5.94 s. Under the same protocol overhead, the average neighbor discovery time in scenario 2.3 is longer than that in scenario 2.2. Scenario 2.3 uses the same width of scanning beams as scenario 2.2, but the number of nodes is larger, resulting in an increased probability of interference within the range of a single beam and a longer neighbor discovery time. The average link survival time of the proposed method in frame setting 2 of scenario 2.3 is 34.17 s, while the corresponding average link survival time in scenario 2.2 is 29.15 s. In scenario 2.2, it is easier to form a bidirectional link. In the case of interference, the bidirectional link is disconnected, and the link survival time is reduced. Comparing Figure 13 with Figure 14, when the number of nodes increases in a fixed network range, the neighbor discovery time and link survival time both increase correspondingly, while the accumulated link offline time changes a little. As shown in Table 11, the average number of undiscovered nodes in scenario 2.3 is much larger than that in scenario 2.2. The interference in scenario 2.3 is more severe and may cause a particular link to fail to build throughout the simulation time.

In Scenario 3.1, a high mobility node within a large coverage area uses the narrowest beam to transmit–receive signals using 3D beam scanning. As shown in Figure 15, the neighbor discovery time of the proposed method is much shorter than that of the comparison method. This is because, for a narrow beamwidth and sparse node distribution, the probability of beam alignment with the random scanning method is low. As a result, the neighbor discovery time takes a long time, and there is a certain probability that some nodes cannot be discovered. In terms of link survival time, the proposed method is much longer than the comparison method; the reason for this includes the following three items: the neighbor discovery time is shorter, the narrow beam greatly reduces the interference, and the discovered nodes always maintain robust connections. Similarly, the accumulated link offline time of the proposed method is shorter than that of the comparison method. With the increase in T, the neighbor discovery time and the accumulated link offline time of the proposed method increase, but the link survival time changes little. As a result, the reconnection time after the link is disconnected increases, and the total link disconnection time increases. The link survival time of the proposed method is about 800 s in the four frame formats, accounting for 80% of the whole simulation time, which shows that once the link is established, the probability of disconnection of the proposed method stays low.

Compared with scenario 3.1, scenario 3.2 narrows the network coverage, increases the number of nodes, and decreases node mobility. Compared with the comparison method, the neighbor discovery time of the proposed method is shorter, but the link survival time is also shorter. The reason for this is similar to scenario 2.1, that is, the proposed method is more likely to form a bidirectional link, and the topology changes lead to tracking failure, which makes the link disconnected and reduces the link survival time. In terms of accumulated link offline time, the proposed method is shorter than the comparison method because the proposed method can rediscover and retrack neighbors soon after link disconnection. With the increase in T, the neighbor discovery time, link survival time, and accumulated link offline time of the proposed method all increase. As can be seen in Table 11, in scenarios 3.1 and 3.2, with large coverage area and high maneuverability, the proposed method offers a more adequate neighbor discovery than the comparison method, and the average number of undiscovered neighbors does not increase with the increase in T. This is because the nodes are widely distributed, and random scanning cannot traverse the complete sphere in a short period of time.

In scenario 3.3, compared with scenario 3.2, the number of nodes becomes bigger, and the network scale and node mobility are reduced to a certain extent. Using the same parameters, the neighbor discovery time and the link survival time of the proposed method are greater than those of the comparison method, while the accumulated link offline time does not vary much. Factors contributing to this phenomenon include wide beam scanning and the movement within a small area, making the neighbor discovery efficiency of the proposed method inferior to that of the comparison method, so the neighbor discovery time is longer. Similar to the situation in scenario 2.1, the comparison method is more likely to form a bidirectional link. When interfered with, the bidirectional link is disconnected successively, resulting in a shorter link survival time of the comparison method. With the increase in T, both the neighbor discovery time and link generation time of the proposed method increase. Compared with scenarios 3.1 and 3.2, there is a certain probability that the proposed method cannot find all neighbors in scenario 3.3, as shown in Table 11, for the same reasons as in scenarios 2.2 and 2.3.

## 6. Discussion

Based on the simulation results in this paper, the following conclusions can be drawn:

1. The protocol proposed in this paper is used to provide multiple access capabilities for phased array directional communication systems and has the advantages of no center node, dynamic resource allocation, high efficiency, and reliability. It can be used in 3D scenarios, such as UAV networking and ground-to-air accessing, and in 2D scenarios, such as mobile vehicle networking.
2. The value of parameter T in the proposed protocol affects indicators such as the neighbor discovery time, link survival time, cumulative link downtime, protocol overhead, and average number of undiscovered nodes. This parameter is related to beamwidth, node mobility, and the minimum distance between the transmitter and receiver. When T is larger than the tracking period, the link lifetime can be still reliably as long as the relative mobility is weak enough. A smaller T can handle the strong relative mobility situation, but the protocol overhead increases accordingly. The appropriate T should be selected according to the actual scenario requirements.
3. The selection of the beamwidth in the proposed protocol is also important. A wider beamwidth can reduce the number of beams needed for each round of neighbor discovery scanning, helping to reduce the neighbor discovery overhead. However, the wider the beamwidth, there may be more neighboring nodes within the beam coverage area, so the collision probability will increase, and more scanning rounds are needed to discover all neighbors successfully. On the other hand, the wider the beamwidth, the shorter the one-hop communication distance, and, accordingly, the number of hops required for end-to-end transmission may increase, making data transmission delay, effective throughput, and other indicators decrease accordingly. Therefore, it is necessary to select the beamwidth according to the actual scenario requirements.

Based on the simulation results in Figure 10, Figure 11, Figure 12, Figure 13, Figure 14, Figure 15, Figure 16 and Figure 17 and Table 11, the proposed protocol is more suitable for scenes with high node mobility, a large network scale, a sparse node distribution, and narrow beamwidths, such as scenario 3.1. However, in scenes with low node mobility, a small network scale, a dense node distribution, and wide beamwidths, such as scenarios 2.1 and 3.3, the proposed protocol is more in favor of link survival time.

Future work will focus on the following aspects:

1. Distributed backoff and probabilistic response methods for neighbor discovery will be considered in order to improve the success rate of neighbor discovery.
2. The design of the data reservation strategy will be improved by making full use of data transmission opportunities brought by neighbor tracking. The supporting mechanism for different types of services will also be used by frame aggregation and multi-step reservation.
3. The impact of accumulated link offline time, disconnection frequency, link survival time, and other parameters on the reliability of data transmission will be studied. Methods such as frequency hopping, adaptive modulation, and adaptive tracking frequency will be used in tracking slots to alleviate the interference among neighboring links.
4. The angle measuring function provided by the phased array antenna will be introduced to provide relative positioning ability in a GPS-denial environment.

## Figures and Tables

**Table 1:** List of acronyms.

Acronym	Description
UAV	unmanned aerial vehicle
FANET	flying ad hoc network
MANET	mobile ad hoc network
ID	identification
3D	three-dimensional
2D	two-dimensional
TDMA	time division multiple access
MAC	medium access control
GNSS	global navigation satellite system

**Table 2:** List of symbols.

Symbol	Definition
θ	The azimuth beamwidth
φ	The elevation beamwidth
$n_{1}$	The number of scanning rounds in a neighbor discovery period
$n_{2}$	The number of beams in each scanning round
T	The duration of the frame
$T_{found}$	The duration of the discovery phase in a frame
$T_{track}$	The duration of the tracking phase in a frame
$T_{transmission}$	The duration of the data transmission phase in a frame
$T_{f\_slot}$	The duration of the discovery slot
$T_{t\_slot}$	The duration of the tracking slot
$T_{d\_slot}$	The duration of the data transmission slot
$T_{slot}$	The duration of the general slot
n	The number of discovery slots in a frame
r	The number of tracking slots in a frame
k	The number of data transmission slots in a frame
z	The number of subframes in a frame
$c_{m}$	The maximum number of neighboring nodes

**Table 3:** Transmitting and receiving state in each scanning round for different IDs (network scale ≤ 3, n1=3).

Node ID	Transmitting and Receiving State
1	001
2	010
3	100

**Table 4:** Transmitting and receiving state in each scanning round for different IDs (network scale ≤ 6, n1=4).

Node ID	Transmitting and Receiving State	Node ID	Transmitting and Receiving State
1	0011	4	1010
2	0110	5	1010
3	1100	6	1001

**Table 5:** Transmitting and receiving state in each scanning round for different IDs (network scale ≤ 10, n1=5).

Node ID	Transmitting and Receiving State	Node ID	Transmitting and Receiving State
1	00011	6	01100
2	00101	7	10001
3	00110	8	10010
4	01001	9	10100
5	01010	10	11000

**Table 6:** n1 required for different network scales.

Network Scale (The Maximum Number of Nodes)	n1
3	3
6	4
10	5
20	6
35	7
70	8
126	9
252	10
……	……

**Table 7:** Simulation scenario descriptions.

Scenario Number	Scenario Description	Configuration				
		Node Count	Node Mobility	Distribution Range	Distribution Characteristics	
1.1	Simple networking among stationary ground vehicles	5	-	10 km × 10 km	Some nodes are clustered, a few nodes are dispersed, the height difference between nodes is ≤10 m, and the minimum distance between nodes is ≥50 m.	
1.2	Typical networking among stationary ground vehicles	10				
2.1	Networking among mobile ground vehicles in a company	10	20~80 km/h	5 km × 5 km	All nodes maneuver arbitrarily, the height difference between nodes is ≤10 m, and the minimum distance between nodes is ≥50 m.	
2.2	Networking among mobile ground vehicles in a battalion	20		20 km × 20 km		
2.3	Networking among mobile ground vehicles in multiple battalion	35		20 km × 20 km		
3.1	Networking among ultra-high speed fixed-wing UAVs	5	300~800 km/h	100 km × 100 km	All nodes maneuver arbitrarily.	The minimum distance between nodes is ≥1000 m.
3.2	Networking among unmanned helicopters and fixed-wing UAVs	10	100~300 km/h	50 km × 50 km		The minimum distance between nodes is ≥500 m.
3.3	Networking among rotary-wing UAVs	20	20~100 km/h	10 km × 10 km		The minimum distance between nodes is ≥50 m.

**Table 8:** The maximum tracing periods required for each scenario.

Scenario Number	Maximum Tracking Period		
	@6° × 9°	@12° × 18°	@24° × 36°
1.1/1.2	Static state, no tracking period limit		
2.1/2.2/2.3	0.1119 s	0.2129 s	0.3673 s
3.1	0.2238 s	0.4259 s	0.7746 s
3.2	0.2984 s	0.5678 s	1.0327 s
3.3	0.0895 s	0.1703 s	0.3098 s

**Table 9:** Multi-access protocol parameters in each scenario. The number of beams in each scanning round, n2, is equal to the number of beams in each scanning plane multiplied by the number of scanning planes in a scanning round, and they are all related to the beamwidth. For scenario 3.1, the beamwidth is θ×φ=6°×9°, n2=360°/θ×180°/φ=60×20=1200.

Scenario Number	T	$T_{slot}$	n	r	k	n1	n2@6° × 9°	n2@12° × 18°	n2@24° × 36°
1.1	500 ms	500 μs	10	10	980	4	-	-	15(15 × 1)
1.2	500 ms	500 μs	20	20	960	5	-	-	* 16(16 × 1)
2.1	300 ms	500 μs	20	20	560	5	-	-	* 16(16 × 1)
2.2	200 ms	500 μs	40	40	320	6	-	** 40(40 × 1)	-
2.3	210 ms	500 μs	70	70	280	7	-	30(30 × 1)	-
3.1	200 ms	1000 μs	10	10	180	4	1200(60 × 20)	-	-
3.2	500 ms	500 μs	20	20	960	5	-	300(30 × 10)	-
3.3	280 ms	500 μs	40	40	480	6	-	-	*** 80(16 × 5)

* The azimuth beamwidth is θ=24°, 360°/θ=15. We set the number of beams in a scanning plane as 16 so that the number of discovery slots in a super-frame 16 × 5 = 80 is divisible by 20, which is the number of subframes in a frame. ** The azimuth beamwidth is θ=12°, 360°/θ=30. We set the number of beams in a scanning plane as 40 so that the number of discovery slots in a super-frame 40 × 6 = 240 is divisible by 40, which is the number of subframes in a frame. *** The azimuth beamwidth is θ=24°, 360°/θ=15. We set the number of beams in a scanning plane as 16 so that the number of discovery slots in a super-frame 16 × 5 × 6 = 480 is divisible by 40, which is the number of subframes in a frame.

**Table 10:** T values and related protocol overheads of four frame settings in each scenario.

Scenario Number	Frame Setting 1		Frame Setting 2		Frame Setting 3		Frame Setting 4	
	T	Overhead	T	Overhead	T	Overhead	T	Overhead
1.1	250 ms	4%	500 ms	2%	1000 ms	1%	2000 ms	0.5%
1.2	250 ms	8%	500 ms	4%	1000 ms	2%	2000 ms	1%
2.1	150 ms	13.33%	300 ms	6.67%	600 ms	3.33%	1200 ms	1.67%
2.2	100 ms	40%	200 ms	20%	400 ms	10%	800 ms	5%
2.3	105 ms	66.67%	210 ms	33.33%	420 ms	16.67%	840 ms	8.33%
3.1	100 ms	20%	200 ms	10%	400 ms	5%	800 ms	2%
3.2	250 ms	8%	500 ms	4%	1000 ms	2%	2000 ms	1%
3.3	140 ms	28.57%	280 ms	14.29%	560 ms	7.14%	1120 ms	3.57%

**Table 11:** The average number of undiscovered nodes under the four frame settings of the two methods in each scenario.

Scenario Number	Method	Frame Setting 1	Frame Setting 2	Frame Setting 3	Frame Setting 4
1.1	proposed method	0.32	0.52	0.42	0.68
	comparison method	0.04	0	0	0.16
1.2	proposed method	2.70	2.72	3.08	3.64
	comparison method	0.06	0.18	0.60	0.74
2.1	proposed method	0	0	0.05	0
	comparison method	0	0	0	0
2.2	proposed method	0.30	0.8	1.00	1.30
	comparison method	0	0	0.10	0.65
2.3	proposed method	4.00	4.94	5.68	7.31
	comparison method	0.14	0.34	0.68	1.88
3.1	proposed method	0	0	0	0
	comparison method	0.48	0.72	1.52	1.92
3.2	proposed method	0	0	0	0
	comparison method	0.25	0.75	1.35	1.85
3.3	proposed method	0.10	0.20	0.35	0.55
	comparison method	0	0	0	0

## Data Availability

The data presented in this study are available on request from the corresponding author due to privacy.
